# Biomarkers in maternal mental health research in Indonesia: toward a pragmatic hybrid framework in low-resource settings

**DOI:** 10.3389/fpsyt.2026.1796186

**Published:** 2026-09-10

**Authors:** Siti Khuzaiyah, Rini Kristiyanti, Gina Anindyajati, Rafael A. Caparros-Gonzalez

**Affiliations:** 1 Midwifery Program, Faculty of Health Sciences, Universitas Muhammadiyah Pekajangan, Pekalongan, Central Java, Indonesia; 2 Dept. of Psychiatry, Cipto Mangunkusumo National General Hospital, Jakarta, Indonesia; 3 Department. of Psychiatry, Faculty of Medicine, Universitas Indonesia, Jakarta, Indonesia; 4 Instituto de Investigación Biosanitaria ibs., Granada, Spain; 5 Facultad de Ciencias de la Salud, Departamento de Enfermería, Universidad de Granada, Granada, Spain

**Keywords:** biomarkers, cortisol, HPA axis, Indonesia, inflammation, low-resource settings, maternal mental health, perinatal depression

## Introduction

Maternal mental health has become an emerging issue recently, including in Indonesia. National data indicate that the prevalence of postpartum depression (PPD) among young mothers (15–24 years) was 4.0%, with higher prevalence observed in urban settings (1). At the same time, broader Indonesian perinatal mental health estimates are relatively high (15.19%–26.15%) compared with those in Malaysia and Singapore, although methods and samples vary (2).

Depression and anxiety during pregnancy and the postpartum period affect a substantial proportion of women worldwide and are associated with adverse outcomes for both mothers and infants. In LMICs, an estimated 15.5–23.0% of women experience perinatal depressive symptoms, with higher prevalence among socioeconomically disadvantaged populations (3, 4). In addition, studies indicate that maternity blues are strong predictor of later postpartum depressive symptoms (5, 6), and untreated maternal depression has been linked to infant developmental delays, bonding, breastfeeding, maternal role, and maternal health-promoting lifestyle during pregnancy (7–9). Consistent with this broader pattern, studies from Indonesia indicate that perinatal mental health disorders remain an important public health concern.

In Indonesia, maternal mental health remains an under-recognised public health issue despite growing awareness among researchers and policymakers. Screening for psychological distress in pregnancy and the postpartum period typically relies on self-report questionnaires such as the Edinburgh Postnatal Depression Scale (EPDS) or the Patient Health Questionnaire (PHQ-9). These tools are widely used since they are inexpensive, easy to administer, and feasible for implementation in primary health care settings, including services delivered by midwives and community health workers (9–12).

Despite the availability of validated screening tools, implementing maternal mental health screening in routine healthcare settings remains challenging, as healthcare workers continue to face barriers to effective screening implementation (13). Nevertheless, questionnaires such as the Edinburgh Postnatal Depression Scale (EPDS) and the Patient Health Questionnaire-9 (PHQ-9) remain the cornerstone of maternal mental health assessment because they capture the subjective emotional and psychological experiences that are fundamental to psychiatric diagnosis.

Rather than replacing questionnaire-based assessments, biological measures should be considered complementary tools that provide objective information on the physiological processes underlying maternal mental health. This complementary role is particularly important because pregnancy and the postpartum period are characterized by dynamic biological and psychological changes that cannot be fully captured through self-report alone (14–16). During these periods, immune and endocrine systems undergo substantial physiological adaptations, resulting in fluctuations in biomarkers across gestation and postpartum. For example, cytokine and C-reactive protein (CRP) trajectories vary according to gestational stage (17). Such physiological changes provide objective information that complements the subjective data obtained from questionnaires.

Furthermore, self-report measures have inherent limitations because pregnancy is also associated with changes in cognition and memory. Pregnant women commonly report subjective memory changes during pregnancy (18), and poorer self-reported memory has been associated with inadequate sleep, higher levels of anxiety, and greater depressive symptoms (19). Consequently, integrating biomarkers with validated questionnaires may provide a more comprehensive assessment of maternal mental health by combining subjective experiences with objective physiological indicators.

Evidence from Indonesia supports the use of validated questionnaire-based screening tools for maternal mental health, although additional complementary approaches are needed to strengthen assessment and interpretation. In the Indonesian sociocultural context, mental health problems during the perinatal period are often framed as moral, spiritual, or familial issues rather than a health condition. This condition may shape how screening results are interpreted and acted upon. Psychometric work in Indonesia has shown that the EPDS can be structurally evaluated and adapted; for instance, an Indonesian EPDS version for pregnant women showed a three-factor structure and a meaningful prevalence signal (20). Local psychosocial research also demonstrates how contextual aspects relate to PPD (21). Experts in Indonesia have already published a consensus to encourage the government to provide sufficient resources to screen, diagnose, and treat mental health disorders, especially in maternal mental health services (22). What remains limited in Indonesia and many other low-resource settings is context-specific evidence regarding whether selected biological markers can provide complementary mechanistic information when interpreted alongside psychosocial measures.

Despite the benefit of biomarkers for illness screening, a recent study indicates that challenges in biomarker utilisation include the complexity of biological samples, low abundance of biomarkers, variability across populations, and difficulties with reproducibility and large-scale validation, while inconsistent findings and reliance on single biomarkers may oversimplify the multifactorial nature of complex diseases (23). Thus, biomarkers should not be viewed as replacements for psychological screening instruments or clinical assessment. Instead, they may serve as research tools to clarify biological pathways linking psychosocial stress to maternal mental health outcomes, including in Indonesia.

Direct generalisation to Indonesia should be done with caution, given the majority of biomarker studies have been carried out in high-income nations. Biomarker distributions and their association with psychological symptoms may be impacted by variations in ethnicity, dietary state, infectious disease load, environmental exposures, socioeconomic difficulties, and access to healthcare. Therefore, rather than being directly transferable, international findings should be seen as hypothesis-generating, highlighting the necessity of locally created longitudinal evidence. This paper discusses key barriers and emerging opportunities for integrating feasible biomarkers into maternal mental health research in Indonesia. Drawing on evidence from global and LMIC contexts, we propose a pragmatic hybrid research approach that combines validated questionnaires with a limited number of minimally invasive biological indicators.

## Heterogeneity and limitations of current biomarker evidence

Biomarker is defined as *‘as a defined characteristic that is measured as an indicator of normal biological processes, pathogenic processes, or responses to an exposure or intervention, including therapeutic interventions*’ (24). Biomarkers include diagnostic biomarkers, monitoring biomarkers, pharmacodynamic/response biomarkers, predictive biomarkers, prognostic biomarkers, safety biomarkers, and Susceptibility/risk (25). Another biomarker is mechanistic biomarker, a biological marker that is directly involved in the underlying pathophysiological processes of a disease. Rather than merely reflecting the presence or consequences of disease, mechanistic biomarkers are embedded within the causal pathways that contribute to disease development (26). In maternal mental health research, biomarkers are objectively measured biological traits that represent physiological processes, pathological processes, or reactions to environmental and psychosocial exposures. However, the results are still inconsistent, despite the fact that many biomarkers have been linked to prenatal anxiety and depression. Biological sample complexity, poor biomarker abundance, patient population heterogeneity, and problems with reproducibility and validation in large and diverse samples are some of the challenges that are mentioned (27). Reliance on a single marker oversimplifies complicated conditions, and biomarkers are sometimes inconsistent. Therefore, rather than being used as stand-alone screening or diagnostic tools, current data favours using biomarkers mainly for mechanistic and exploratory study.

## Structural barriers to biomarker research in low-resource settings

In LMIC contexts, mainly in Indonesia, several structural barriers limit biomarker research.

### Research funding and health system priorities

In low-resource settings, limited funding is a significant barrier to biomarker utilisation due to the need for bulky, expensive instrumentation. Biomarker assays (Enzyme-Linked Immunosorbent Assay (ELISA) kits, multiplex panels, cold-chain requirements) can be unaffordable, especially when multiple or repeated sampling is required (28, 29). Health systems may prioritise visible physical outcomes such as maternal mortality, stunting, and infections, rather than “non-physical” outcomes such as mental health and biology. Thus, questionnaires become the default since they are cheaper and can be scaled fast (30, 31).

### Laboratory capacity and infrastructure

Indonesia is geographically diverse, and laboratory capacity is uneven. Many districts lack reliable immunoassay facilities, quality control systems, or trained technicians. Even when university labs exist, they may be far from community sites, causing delays and reducing feasibility for time-sensitive biomarkers (e.g., some cytokines). Limited equipment maintenance and reagent stockouts also affect continuity of biomarker utilisation. Studies conducted during COVID-19 reflected these conditions (32, 33). However, several infrastructures in Indonesia might be helpful for the biomarker research while strengthening community-based research, such as Integrated Biomedical Laboratory Ministry of Health, National Research and Innovation Agency (BRIN), University based laboratory, and the existing maternal cohorts and Posyandu/Puskesmas networks.

### Logistical challenges in biological sampling

Logistics is not only about distance, but it is also about stability. Many biomarkers require controlled temperature and rapid processing (34). Delayed transport can degrade samples and introduce measurement error (35). Reagents and consumables utilities can be hard to access, arrive late, and become costly due to import dependence. These realities make biomarker research less attractive to researchers in low-resource settings and to busy maternal health services.

### Physiological variation during pregnancy

Pregnancy itself is a confounder. Immune markers and CRP change across gestation and postpartum, following characteristic trajectories (17). Cortisol also changes substantially in pregnancy, and the “normal” pattern differs by trimester and method (36). If we collect one sample without standardized timing, there will be a risk of misclassification (e.g., interpreting regular late-pregnancy endocrine shifts as “pathological”). This is a technical challenge that becomes even more pronounced when low resource constraints limit repeat sampling. Since physiological trajectories vary significantly across trimesters and the postpartum period, single cross-sectional data may be deceptive. Therefore, it is better to use longitudinal sampling with reference ranges unique to gestational age. Potential confounding variables like obesity, infection, medication use, dietary status, sleep quality, socioeconomic adversity, and obstetric complications must also be considered.

### Ethical governance and community trust

Biomarker collection requires more complex consent, such as the type of sample, future use, storage duration, data linkage, and potential sharing/export. Stakeholder research from other Low and Middle-Income Countries (LMIC) contexts highlights concerns around trust, fairness, ownership, and governance when biological samples are stored or transferred (37). Biomarker-based approaches also raise concerns regarding biological determinism. Overemphasis on biological mechanisms may inadvertently reinforce stigma by portraying maternal mental disorders as fixed biological abnormalities and may reduce attention to social determinants such as poverty, domestic violence, and gender inequities. Biomarker findings should therefore be interpreted within a biopsychosocial framework and communicated carefully to avoid discrimination and labelling. In Indonesia, these concerns must be managed carefully since maternal populations may experience stigma related to mental health, and communities may be cautious about blood or genetic studies. Trained human resources and SOPs are needed to ensure confidentiality, culturally sensitive consent, and safe handling, yet these are often limited.

### Sociocultural aspects of maternal mental health problems & services

Although many results from maternal mental health biomarker research hold significant potential to provide complementary biological information for mechanistic research, there is as yet no clinically useful biomarker in commercial development for any maternal mental health problems (38). Adding the strong stigma surrounding mental illness in Indonesian communities may raise concern, whether a biological marker would inadvertently reinforce labelling and social exclusion rather than help seeking. In terms of the meaning of illness, the relationship between healthcare workers and mothers in Indonesia still follows a hierarchical order. Thus, biomarker-based screening may shift decision-making further away from women’s own narratives and lived experiences (39), further marginalising subjective experiences and the already underrepresented women’s voice in clinical encounters.

Taken together, these difficulties demonstrate why careful planning and context-appropriate approaches are necessary for biomarker research in LMIC settings rather than direct clinical application. Involving women and families, working with midwives and Posyandu cadres, community advisory boards, feedback systems, and open communication about sample storage are all ways to improve community engagement. Instead of acting as locations for biomarker-based screening, Puskesmas and Posyandu might offer chances for participant recruitment and long-term monitoring.

## Opportunities for feasible biomarkers in maternal mental health research

Despite these structural barriers, selected biomarkers may still provide valuable insights into biological pathways associated with maternal mental health (40). Rather than relying on complex laboratory panels, researchers in low-resource settings may prioritise feasible, minimally invasive indicators compatible with community-based research. However, the same scientific standards regarding validity, reproducibility, and external validation apply regardless of setting.

### HPA axis and stress hormones

Cortisol (serum/salivary/hair/nail), placental corticotropin-releasing hormone (pCRH), and dexamethasone suppression tests are widely used to assess stress-system dysregulation in perinatal depression. Studies usually report altered cortisol dynamics (e.g., higher late-pregnancy cortisol or blunted postpartum HPA response) in women with perinatal depressive symptoms. Still, patterns vary depending on gestational stage and method. Among the available biological matrices, salivary cortisol provides a practical and non-invasive measure of hypothalamic-pituitary-adrenal (HPA) axis activity that is particularly suitable for stress research. However, its interpretation requires careful consideration of biological and methodological sources of variability, including estrogen levels, medical conditions, and standardized sampling procedures (41).

Among these methods, hair cortisol concentration (HCC) has gained increasing attention as an indicator of chronic stress exposure. Research indicates that Hair cortisol has shown potential associations with postpartum depressive symptoms in some studies (42–44), although findings remain inconsistent and require replication across diverse populations, including LMIC settings. Hair cortisol reflects cumulative cortisol secretion over several months and can be obtained through a small hair sample from the scalp (45). These characteristics make hair cortisol particularly suitable for population studies conducted in resource-constrained environments (46).

On the other hand, salivary cortisol provides another feasible method for assessing acute stress responses. Collecting Saliva is non-invasive and can be performed by untrained phlebotomists, making it suitable for community-based research settings. Given awareness of potential sources of variance that may affect this measure, salivary cortisol is considered a useful biomarker in stress-related studies (41).

Another sample is taken from nail. Nail cortisol elevation was associated with chronic stress exposure and depression (47). Fingernails collection was an easily conducted, well-tolerated application to evaluate stress markers, make it possible to be conducted in low-income settings.

### Inflammatory markers

Immune dysregulation is commonly observed in individuals with posttraumatic stress disorder (PTSD), and this immune response is partly influenced by variation in the highly polymorphic human leukocyte antigen (HLA) locus, which has also been linked to major psychiatric conditions such as schizophrenia, major depression, and bipolar disorder (48).

In maternal populations, psychological factors are associated with altered immune responses, potentially contributing to adverse pregnancy outcomes. Higher well-being was associated with lower leptin levels in late pregnancy, while greater stress and increased risk of depression were linked to reduced pro-inflammatory markers (TNF-α, ICAM1, IL-17A) throughout pregnancy (49).

### Neurotrophic and neuropeptide markers

Brain-derived neurotrophic factor (BDNF) is one of the most studied neurotrophic biomarkers. A study revealed that serum BDNF decreases across pregnancy and rises postpartum, with lower levels in the second and third trimesters predicting greater depressive symptoms and lower birth weight. In comparison, Black women show higher BDNF levels than White women (50).

In addition, oxytocin (plasma or serum) has been repeatedly investigated. Studies suggesting that lower late-pregnancy or early-postpartum oxytocin, or a decline across this window, may indicate greater postpartum depression risk (51, 52), though overall findings remain mixed.

It is important to emphasise that these biomarkers should not stand alone as a single screening; their primary value lies in research exploring interactions between biological stress responses and psychosocial determinants of maternal mental health.

The comparison between each potential biomarkers can be found in **Table 1**.

**Table 1:** Candidate biomarkers for maternal mental health research in low-resource settings.

Biomarker	Biological relevance	Sampling method	Relative cost	Feasibility in Indonesia	Major limitations	Suggested references
Hair cortisol	Chronic stress exposure and long-term HPA-axis activity	Hair sample	Low	High	Hair treatment, ethnicity, gestational variation, lack of standardized reference ranges	(42, 45, 46, 53, 54)
Nail cortisol	Cumulative chronic stress and HPA-axis activation over weeks to months	Fingernail clipping	Low	High	Limited validation studies; influenced by age, sex, ethnicity, nail growth and cosmetic factors	(41, 47, 55)
Salivary cortisol	Acute stress response and HPA-axis reactivity	Saliva	Low	High	Strong circadian variation, timing-sensitive collection, intra-individual variability	(56, 57)
Placental corticotropin-releasing hormone (pCRH)	Placental stress signaling and neuroendocrine adaptation during pregnancy	Maternal blood	Moderate to high	Moderate	Marked gestational-age dependence and laboratory requirements	(58, 59)
hsCRP	Marker of systemic inflammation and low-grade immune activation associated with depressive symptoms. Can be used as long-term monitoring	Serum or dried blood spot (DBS)	Moderate	Moderate to high	Confounding by infection, obesity, pregnancy complications, and gestational age	(60)
Inflammatory cytokines (IL-6, TNF-α, IL-17A)	Immune dysregulation and inflammatory pathways related to stress and depression. Might be used for determining severity in research and for tracking development of the fetus	Serum or plasma	High	Low to moderate	High biological variability, assay complexity, inconsistent findings across studies	(48, 49)
Brain-derived neurotrophic factor (BDNF)	Neuroplasticity, stress adaptation, and neuronal resilience	Serum	Moderate	Moderate	Population differences, gestational variation, inconsistent associations with depressive symptoms	(50)
Oxytocin	Maternal bonding, social behavior, and stress regulation. However, it is more on measuring vulnerabilities/risks rather than the actual event.	Plasma or serum	High	Low	Poor assay reproducibility and mixed findings across studies	(51, 52)

HPA-axis, hypothalamic-pituitary-adrenal axis; pCRH, placental corticotropin-releasing hormone; hsCRP, high-sensitivity C-reactive protein; IL, interleukin; TNF-α, tumor necrosis factor-α; BDNF, brain-derived neurotrophic factor.

## Toward A pragmatic hybrid research approach

Several pragmatic priorities may facilitate the responsible development of biomarker research in low-resource settings such as Indonesia.

First, pilot studies should be embedded within existing maternal health services and prospective cohorts to evaluate the feasibility and acceptability of biological sampling. Candidate biomarkers should remain limited to relatively affordable and minimally invasive approaches, including hair cortisol, salivary cortisol, and dried blood spot measurements of inflammatory markers such as hsCRP.

Second, longitudinal study designs and gestational age-specific sampling strategies should be prioritized to account for the dynamic physiological changes that occur across pregnancy and the postpartum period. Alignment of sample collection with routine antenatal and postnatal visits may improve feasibility while minimizing participant burden.

Third, harmonized protocols and shared standard operating procedures should be established across collaborating institutions to improve reproducibility and facilitate future multicentre studies. Such efforts may eventually support the development of locally relevant reference patterns for Indonesian populations.

Fourth, robust ethical governance and meaningful community engagement should accompany all stages of biomarker research. Transparent consent procedures, culturally sensitive communication, clear policies regarding sample storage and data sharing, and investments in local laboratory capacity are essential to ensure that research remains equitable, culturally appropriate, and socially responsible.

Finally, future studies should prioritize multimodal analytical approaches that integrate biological variables with validated psychosocial assessments rather than seeking single diagnostic biomarkers. Conventional regression models, latent variable approaches, and machine-learning techniques may offer opportunities to explore complex interactions among biological, psychological, and social determinants. Nevertheless, these approaches should remain exploratory and require extensive external validation before any consideration of clinical application.

**Figure 1:**
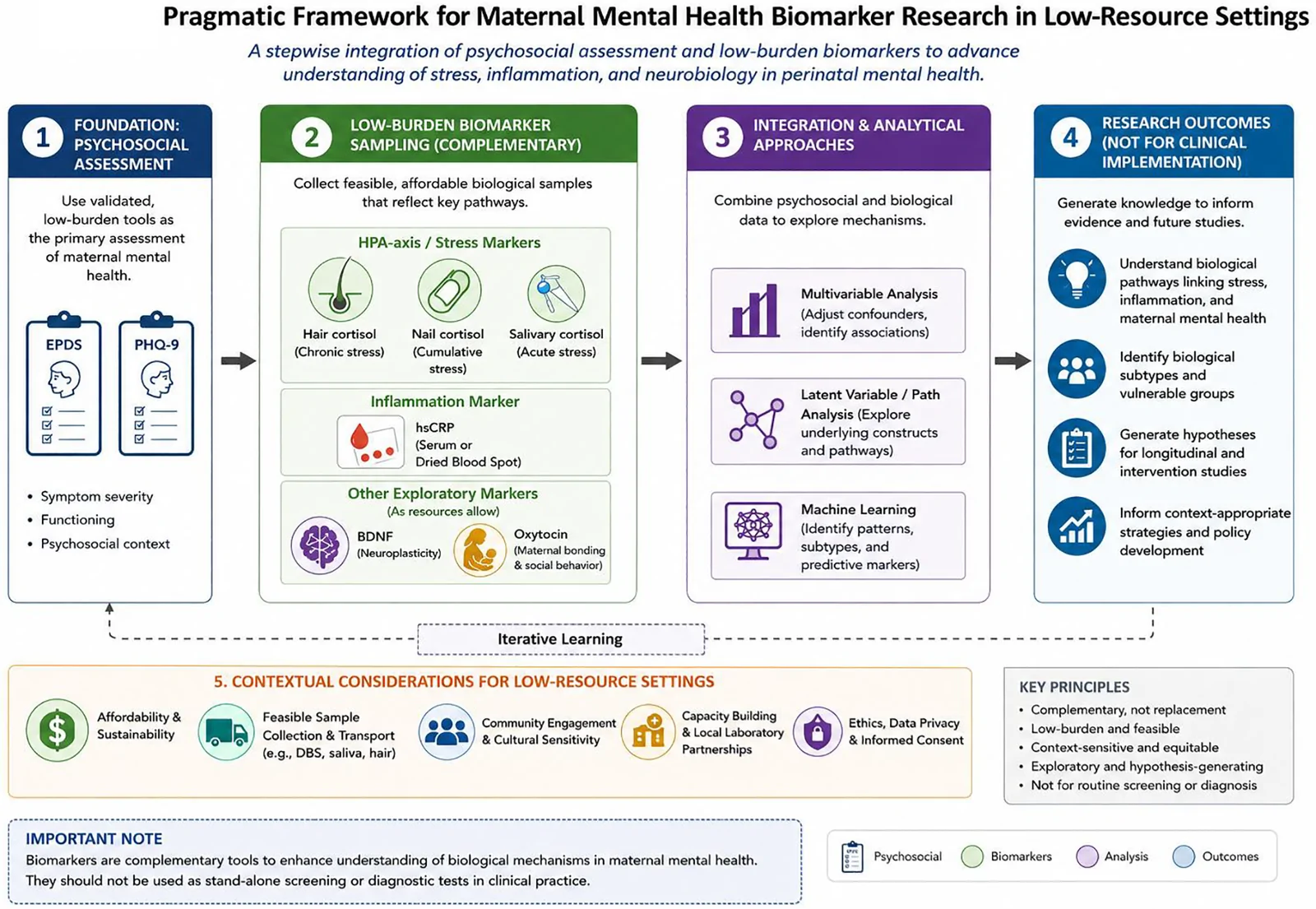
Pragmatic framework for maternal mental health biomarker research in low resources setting.

Overall, a cautious, phased, and context-sensitive approach is likely to provide the most appropriate pathway for advancing maternal mental health biomarker research in Indonesia and other low-resource settings (refer to **Figure 1**).

The framework (**Figure 1**) demonstrates a methodical process for combining inexpensive, minimally intrusive biomarkers with validated psychosocial assessments. Maternal mental health assessment is based on standardised questionnaires like the Edinburgh Postnatal Depression Scale (EPDS) and Patient Health Questionnaire-9 (PHQ-9). Hair cortisol, nail cortisol, salivary cortisol, and hsCRP are examples of complementary biomarkers that may offer more details about inflammatory and stress-related processes. Exploration of intricate relationships between biological, psychological, and environmental aspects may be made easier by integrating psychosocial and biological data using multivariable analyses, latent-variable approaches, and machine-learning techniques.

To improve viability in low-resource contexts, contextual factors like affordability, community involvement, ethical governance, and local laboratory capacity are taken into account. In contrast to routine clinical screening or diagnostic use, the framework highlights that biomarkers are complementary research tools used mainly for exploratory and mechanistic investigations. Furthermore, instead of creating separate systems, biomarker collecting could be incorporated into already-existing prenatal and postpartum services including Community Health Services (*Puskesmas)*, Integrated Health Services (*Posyandu)*, antenatal care (ANC) visits, maternal health programs, and midwife networks. Maintaining quality assurance requires managing SOPs, putting internal quality control into place, applying external quality assurance, guaranteeing calibration standards, improving assay repeatability, maintaining sample transportation, and assessing laboratory proficiency.

## Conclusion

This paper highlights that biomarkers should be regarded as complementary to, rather than replacements for, validated psychosocial assessments and clinical evaluation in maternal mental health. While questionnaires remain the cornerstone of screening and diagnosis because they capture women’s subjective psychological experiences, biomarkers may provide additional objective information on the biological processes associated with maternal mental disorders.

Current evidence on endocrine, inflammatory, and neurotrophic biomarkers remains heterogeneous and frequently contradictory. Differences in study populations, biological matrices, timing of sample collection, laboratory methods, and the physiological adaptations that occur throughout pregnancy and the postpartum period have limited the reproducibility and comparability of findings. Consequently, no individual biomarker has demonstrated sufficient consistency, diagnostic accuracy, or predictive performance to support routine clinical use for screening, diagnosis, or treatment monitoring of perinatal mental disorders.

At present, the primary value of biomarkers lies in mechanistic research and hypothesis generation. When interpreted within a biopsychosocial framework and integrated with psychosocial and environmental factors, biomarkers can improve understanding of the biological pathways linking stress, inflammation, and maternal mental health, but they are not yet clinically actionable.

For Indonesia, where validated psychosocial screening tools are increasingly available but implementation remains challenging, future research should prioritize longitudinal studies that integrate biological markers with psychosocial assessment using harmonized and culturally sensitive methodologies. Such evidence might improve understanding of maternal mental health within the local sociocultural context and inform whether selected biomarkers have a meaningful complementary role in clinical assessment.
